# A case series of maturity-onset diabetes of the young highlighting atypical presentations and the implications of genetic diagnosis

**DOI:** 10.20945/2359-4292-2023-0239

**Published:** 2024-07-30

**Authors:** Meghana Narasimhegowda, Vani Hebbal Nagarajappa, Raghupathy Palany

**Affiliations:** 1 Division of Pediatric and Adolescent Endocrinology Indira Gandhi Institute of Child Health Bengaluru India Division of Pediatric and Adolescent Endocrinology, Indira Gandhi Institute of Child Health, Bengaluru, India

## Abstract

Maturity-onset diabetes of the young (MODY) is a clinically heterogeneous group of monogenic diabetes characterized by onset at a young age and an autosomal dominant mode of inheritance. Notably, MODY accounts for 2%-5% of all diabetes cases, and its distinction from types 1 (T1DM) and 2 (T2DM) diabetes mellitus is often challenging. We report herein the cases of two girls and a boy who presented initially with diabetic ketoacidosis. In view of the strong family history of diabetes in all three of them, the diagnosis of MODY was considered and confirmed by molecular testing. The patient in Case 1 (a 10-year-old girl) had a variation in the HNF1A gene (MODY 3). The patient in Case 2 (a 13-year-old girl) had a variation in the HNF1B gene (MODY 5) and was also clinically diagnosed with HNF1B MODY due to short stature, abnormal renal function, renal cysts, unicornuate uterus, and diabetic ketoacidosis at presentation. The patient in Case 3 (a 14-year-old boy) had a variation in the KCNJ11 gene (MODY 13) and presented with diabetic ketoacidosis; after initially being treated as having T1DM, he developed progressive weight gain, acanthosis nigricans, and decreased requirement of insulin. The patients in Cases 1 and 3 were subsequently treated with oral sulfonylureas and insulin was gradually tapered and interrupted, resulting in drastic improvement in glucose control. The patient in Case 2 remained on insulin, as this is the appropriate management for MODY 5. This case series demonstrates that atypical cases of MODY with ketoacidosis do occur, underscoring the potential for this complication within the phenotypic spectrum of MODY. In patients with atypical presentations, a thorough family history taking may reveal the diagnosis of MODY.

## INTRODUCTION

Maturity-onset diabetes of the young (MODY) is a clinically heterogeneous group of monogenic defects in beta‐cell function characterized by onset at young age, nonketotic diabetes that usually does not require insulin, and an autosomal dominant mode of inheritance (1,2). The overall prevalence of MODY among European cohorts is estimated to be 1 per 10,000 adults and 1 per 23,000 children, with about 1%-5% of all cases of diabetes being attributed to this condition (3). Distinguishing MODY from types 1 (T1DM) and 2 (T2DM) diabetes mellitus is often challenging (3). At least 14 different forms of MODY are known to date, of which *HNF1A* MODY is the most common (30%-65%) subtype (4). The *HNF1B* MODY accounts for < 5% of all MODY subtypes, while mutations in *KCNJ11* MODY are very rare and found in < 1% of all MODY cases (1,4). We report herein a case series of three patients with *HNF1A, HNF1B,* and *KCNJ11* MODY who had ketoacidosis at presentation and were initially misdiagnosed as having T1DM.

## CASE REPORTS

### Case 1

Miss P, an adolescent girl aged 10.75 years and the second child born to nonconsanguineous parents, was born at term by normal vaginal delivery with a birth weight of 3.1 kg. Her perinatal period was uneventful and she had no history of hypoglycemia or hyperglycemia. She presented to the emergency department with polyuria and polydipsia of 2 weeks’ duration and complaints of abdominal pain for 2 days. On examination, she had tachycardia and acidotic breathing, and her body mass index (BMI) was 17.12 kg/m^2^ (z-score 0.048). The diagnostic workup revealed hyperglycemia with a random plasma glucose level of 389 mg/dL, glycated hemoglobin (HbA1c) 13.2%, ketonuria (urinary ketone bodies +++), and high anion gap metabolic acidosis (arterial blood gas results: pH 7.12, bicarbonate 8 mEq/L). The patient was diagnosed with T1DM accompanied by diabetic ketoacidosis and was treated according to the guidelines of the International Society of Paediatric and Adolescent Diabetes (ISPAD) until resolution of diabetic ketoacidosis (about 48 hours). Subsequently, she was started on a basal bolus regimen of subcutaneous insulin at a dose of 0.8 units/kg/day (5).

At follow-up, the patient was found to have poor glycemic control (HbA1c 12.4%) despite a good adherence to insulin therapy, an organized meal plan, and regular exercises. Her plasma glucose levels showed great variability, with frequent fluctuations between hypoglycemia and hyperglycemia. On further investigation, she was found to have a C-peptide level of 0.68 ng/mL (with a concomitant blood glucose level of 210 mg/dL and normal renal function), showing a degree of insulin secretion that was not compatible with T1DM. She also had a very low level of glutamic acid decarboxylase (GAD) antibodies (<10 IU/mL).

A detailed family history of hyperglycemia revealed that the proband’s mother had received a diagnosis of diabetes at the age of 35 years and had been on regular treatment with oral hypoglycemic agents (a combination of metformin 500 mg and glimepiride 1 mg twice daily) since then. At diagnosis, the proband’s mother had a BMI of 23.4 kg/m^2^, fasting plasma glucose of 140 mg/dL, 2-hour plasma glucose of 210 mg/dL during oral glucose tolerance test (OGTT), and HbA1c level of 13.3%. The proband’s mother had normal blood pressure with no other metabolic comorbidities. The proband’s maternal grandmother was also treated with the same oral medications as the proband’s mother after she received a diagnosis of diabetes at the age of 45 years, with a fasting plasma glucose of 136 mg/dL and a 2-hour plasma glucose of 220 mg/dL. The BMI of the proband’s maternal grandmother at diagnosis was 24.6 kg/m^2^ and she did not have hypertension or dyslipidemia at that time point. No test results for pancreatic autoantibodies or C-peptide were available. With all these elements combined, the diagnosis of MODY was suspected in the proband’s case.

Seventy-eight genes associated with MODY and other forms of monogenic diabetes were screened and analyzed for pathogenic variants by targeted sequencing analysis using a monogenic gene panel, and direct sequencing by the Sanger method was performed for validation of identified mutations. Molecular testing revealed a novel heterozygous duplication variation *HNF1A*(NM_000545.8):c.1733dupT(p.Gln579AlafsTer70) in the *HNF1A* gene in exon 9, resulting in premature truncation of the protein, known to cause MODY 3. The *p.Gln579AlafsTer70* variation has not been reported in the 1000 Genomes, Exome Aggregation Consortium (ExAC), or gnomAD databases. MutationTaster (http://www.mutationtaster.org) was the online bioinformatics tool to predict that this variation was likely to have a disease-causing effect. Segregation analysis showed that the patient had inherited the variation from her mother (who also had diabetes). This particular variation was not found in her father and sibling (both of whom did not have diabetes). Based on the gathered evidence, this variation can be classified as a “likely pathogenic” variant of the *HNF1A* gene according to the 2015 American College of Medical Genetics and Genomics (ACMG) classification of pathogenicity (6). The patient was then started on oral glibenclamide at 0.6 mg/kg/day (total daily dose 17.5 mg/day) and insulin therapy was gradually withdrawn. At the last follow-up, she was on oral glibenclamide 0.3 mg/kg/day (total daily dose 10 mg) with a good glycemic control (HbA1c at follow-up was 7.2%), as depicted in Table 1.

**Table 1:** Timeline of events in Case 1 (HNF1A MODY)

Timeline	January 2019	July 2019	September 2019	June 2020
Diagnosis	T1DM with DKA	MODY considered	Molecular diagnosis of HNF1A MODY	MODY
C-peptide	N/A	0.68 ng/mL*	N/A	N/A
GAD antibody	N/A	Negative	N/A	N/A
HbA1c	13.2%	12.4%	12.4%	7.2%
Treatment	Insulin 0.8 U/kg/day	Insulin 0.8 U/kg/day	Started glibenclamide 0.6 mg/kg/day (total daily dose 17.5 mg), insulin gradually tapered and stopped	Glibenclamide 0.3 mg/kg/day (total daily dose 10 mg)
Hypoglycemia (PG < 70 mg/dL)	N/A	Frequent	Frequent	No
Hyperglycemia (Pg > 180 mg/dL)	N/A	Frequent	Frequent	No

*Concomitant plasma glucose level 210 mg/dL and normal renal function.

Abbreviations: DKA, diabetic ketoacidosis; HbA1c, glycated hemoglobin; MODY, maturity-onset diabetes of the young; N/A, not available; PG, plasma glucose; T1DM, type 1 diabetes mellitus.

### Case 2

Miss S, an adolescent girl aged 13.25 years, was the firstborn to nonconsanguineous parents. She was born at term by normal vaginal delivery with a birth weight of 2.5 kg. The patient had an uneventful perinatal period, with no past history of hypoglycemia or hyperglycemia. Her family history was strong for diabetes in both parents and maternal grandfather. She presented to the emergency department with complaints of polyuria and polydipsia for 1 month and abdominal pain and vomiting for 2 days. On examination, she was short for her age, with a height of 119 cm (<3rd percentile for age, z-score -5.0), weight of 19 kg (z-score -4.4), and BMI 13.4 kg/m2 (z-score of -2.22). Her diagnostic workup revealed a random plasma glucose level of 302 mg/dL, HbA1c 15.6%, urinary ketone bodies +++, high anion gap metabolic acidosis (pH 7.08, bicarbonate 7 mEq/L), and abnormal renal function (blood urea 78 mg/dL, serum creatinine 2.1 mg/dL). An ultrasound of the abdomen and pelvis showed contracted kidneys bilaterally, a simple renal cyst in the right kidney (measuring about 6 mm), and a unicornuate uterus. Her fasting C-peptide level was 0.3 ng/mL (with a concomitant plasma glucose level of 345 mg/dL) and she tested negative for GAD antibodies (10 IU/mL). She was initially diagnosed with T1DM and diabetic ketoacidosis and her treatment was later changed to a basal bolus regimen of subcutaneous insulin at a dose of 1 unit/kg/day.

A detailed family history revealed that the proband’s mother had presented two spontaneous abortions in the first trimester in previous pregnancies and was diagnosed with overt diabetes (fasting plasma glucose 136 ng/dL and 2-hour plasma glucose 215 mg/dL during OGTT) and hypertension in the first trimester of her third pregnancy at the age of 26 years. The proband’s mother measured 135 cm in height and had a BMI of 26.3 kg/m^2^; she had undergone ultrasound examination of the abdomen and pelvis and, although the report was not available, was told that she had a cyst in her right kidney. Due to financial constraints, the proband’s mother had declined molecular testing and was then started on insulin therapy, which was maintained after pregnancy. Without regular follow-up, the proband’s mother developed kidney failure at the age of 36 years. The proband’s father was found to have hyperglycemia (fasting plasma glucose 148 mg/dL) at the age of 45 years. At the time of diagnosis, the father’s BMI was 28 kg/m^2^ and he had stage 1 hypertension with no other metabolic comorbidities. The proband’s maternal grandfather was also diagnosed with diabetes and hypertension at the age of 30 years and was on treatment with oral antihypertensive and oral hypoglycemic agents for about 1 year before switching to insulin in view of uncontrolled diabetes. No other medical records of the proband’s maternal grandfather were available. Based on the proband’s age at diagnosis (adolescence), a strong family history of diabetes in both parents and maternal grandfather, short stature, presence of renal cyst and uterine malformation (a Faguer (7) [or *HNF1B*] score of 12), and absence of GAD antibodies, the diagnosis of *HNF1B* MODY was suspected.

Molecular testing of the patient revealed a novel heterozygous variation *HNF1B*(NM_000458.4): c.458A>G(p.His153Arg) in the *HNF1B* gene, causing a substitution of amino acid arginine for histidine at codon 153 (exon 2). The variation in the same codon 153 –*HNF1B*(NM_000458.4):*c.457C*>A (p.*His153Asn*) (8) – has been found in patients with MODY 5. Segregation analysis showed that the proband had inherited the mutation from her mother (who also had diabetes). The proband’s father (who also had diabetes) did not harbor this mutation. Based on the gathered evidence, this variation can be classified as a “likely pathogenic” variant of the *HNF1B* gene according to the 2015 ACMG classification of pathogenicity (6). Since hypoplasia of the pancreas and insulin resistance have been described as part of the spectrum of *HNF1B* gene disorders (2), the proband was maintained on insulin therapy, with which she had good glycemic control (HbA1c 6.8% during follow-up). The correct molecular diagnosis in this case was critical to the patient, who could then be maintained on insulin therapy in addition to undergoing follow-up of her early-onset renal dysfunction and electrolyte disturbances characteristic of *HNF1B* MODY. However, 7 years after the diagnosis, the proband progressed to end-stage renal disease (Table 2).

**Table 2:** Timeline of events in Case 2 (HNF1B MODY)

Timeline	May 2014	June 2014	February 2016	January 2021
Diagnosis	T1DM, suspected HNF1B MODY	Molecular diagnosis of HNF1B MODY	HNF1B MODY	HNF1B MODY
Other clinical features	Short stature, abnormal renal function, contracted kidneys bilaterally with a simple renal cyst in the right kidney, unicornuate uterus	N/A	Onset of hypomagnesemia, hypokalemia	N/A
Serum creatinine	1.5 mg/dL	1.4 mg/dL	2.1 mg/dL	3.8 mg/dL
Estimated GFR	32.2 mL/min/1.73 m^2^	34.5 mL/min/1.73 m^2^	22.9 mL/min/1.73 m^2^	14.4 mL/min/1.73 m^2^
CKD stage	Stage 3b	Stage 3b	Stage 4	ESRD
C-peptide	0.3 ng/mL (concomitant PG of 345 mg/dL and serum creatinine 2.1 mg/dL)	N/A	N/A	N/A
GAD antibody	Negative	N/A	N/A	N/A
HbA1c	15.6%	N/A	7.4%	6.8%
Treatment	Insulin 1 U/kg/day	Insulin 1.2 U/kg/day was maintained	Insulin maintained	Insulin 1.2 U/kg/day, started on hemodialysis

Abbreviations: CKD, chronic kidney disease; ESRD, end-stage renal disease; GAD, glutamic acid decarboxylase; GFR, glomerular filtration rate; HbA1c, glycated hemoglobin; MODY, maturity-onset diabetes of the young; N/A, not available; PG, plasma glucose; T1DM, type 1 diabetes mellitus.

### Case 3

Master V, an adolescent boy aged 14.5 years, was the firstborn to nonconsanguineous parents. He was born at term by normal vaginal delivery with a birth weight of 2.15 kg and had an uneventful perinatal period and no past history of hypoglycemia or hyperglycemia. The proband presented to the emergency department with complaints of polyuria, polydipsia, and weight loss for 2 weeks and complaints of abdominal pain and vomiting for 2 days. The diagnostic workup revealed a random plasma glucose level 632 mg/dL, elevated HbA1c of 13.5%, urinary ketone bodies +++, high anion gap metabolic acidosis (arterial blood gas results: pH 6.95, bicarbonate 5.9 mEq/L). He was diagnosed with T1DM and diabetic ketoacidosis and was treated with fluids and insulin infusion as per ISPAD guidelines until resolution of the diabetic ketoacidosis. Subsequently, the proband was started on a basal bolus regimen of subcutaneous insulin at a dose of 1 unit/kg/day.

During the subsequent 1 year, the proband’s insulin requirement gradually decreased to as low as 0.3 unit/kg/day and he started developing multiple episodes of hypoglycemia, even with the low insulin dose. At that time, his HbA1c was 5.6%. Over the next few months, he presented with progressive weight gain, his BMI increased from 19 kg/m^2^ (z-score 0.10) to 21.6 kg/m^2^(z-score of 1.17, indicating overweight). The appearance of acanthosis nigricans (Grade 2) was noted in the cervical region, but the proband’s blood pressure and lipid profile were within normal limits. Insulin treatment was interrupted, and the proband was reevaluated considering a possible diagnosis of T2DM or MODY. His fasting C-peptide level was normal (1.2 ng/mL) and his GAD antibody level was low (10 IU/mL). A detailed family history revealed that both parents and paternal grandmother were diagnosed with diabetes around the fourth decade of life. His father was diagnosed with diabetes at the age of 34 years and was on regular treatment with oral hypoglycemic agents (a combination of metformin 500 mg and glimepiride 1 mg twice daily) since then. At diagnosis, the father’s BMI was 24.4 kg/m^2^, his fasting plasma glucose was 156 mg/dL, 2-hour plasma glucose was 228 mg/dL on OGTT, and HbA1c level was 12%. The father’s blood pressure was within the normal range and he had no other metabolic comorbidities. The proband’s mother was found to have hyperglycemia (HbA1c 11.9%) at the age of 48 years. At the time of diagnosis, the mother’s BMI was 29 kg/m^2^ and she had stage 1 hypertension with no other metabolic comorbidities. The proband’s paternal grandmother was treated with the same oral medications as the proband’s father after she was diagnosed with diabetes at the age of 45 years; she had no hypertension or dyslipidemia at diagnosis, and test results for pancreatic autoantibodies and C-peptide were not available.

Molecular testing revealed a known heterozygous missense mutation *KCNJ11*(NM_000525.4): c.853G>A(p.Val285Ile) in exon 1 of the *KCNJ11* gene that results in substitution of isoleucine for valine at codon 285. This variant is predicted to be damaging by bioinformatics tools such as PolyPhen-2, SIFT, and MutationTaster. The observed variation has been reported previously in patients affected with transient neonatal diabetes mellitus (9). Segregation analysis showed that the patient had inherited the mutation from his father (who also had diabetes). The proband’s mother (who also had diabetes) did not harbor this mutation (Table 3). Based on the evidence gathered, this variant can be classified as a “likely pathogenic” variant of the *KCNJ11* gene according to the 2015 ACMG classification of pathogenicity (6). Hence, the patient was started on oral glibenclamide 5 mg/day (0.08 mg/kg/day) and progressed with good glycemic control on home blood glucose monitoring, with a follow-up HbA1c level of 6.9% (Table 3).

**Table 3:** Timeline of events in Case 3 (KCNJ11 MODY)

Timeline	September 2019	July 2020	September 2020	January 2021
Diagnosis	T1DM	MODY considered	Molecular diagnosis of KCNJ11 MODY	KCNJ11 MODY
Other clinical features	N/A	Weight gain, acanthosis nigricans	Weight gain, acanthosis nigricans	N/A
BMI	19 kg/m^2^	21.6 kg/m^2^	21.6 kg/m^2^	19.6 kg/m^2^
Fasting lipid profile	N/A	Normal	Normal	Normal
C-peptide	N/A	1.2 ng/mL*	N/A	N/A
GAD antibody	N/A	Negative	N/A	N/A
HbA1c	13.5%	5.6%	5.8%	6.9%
Treatment	Insulin 0.8 U/kg/day	Insulin 0.3 U/kg/day	Started on glibenclamide 5 mg/day (0.08 mg/kg/day), stopped insulin, lifestyle modifications	Glibenclamide, lifestyle modifications
Hypoglycemia	N/A	Frequent	Frequent	No

*Concomitant plasma glucose of 215 mg/dL and normal renal function.

Abbreviations: BMI, body mass index; HbA1c, glycated hemoglobin; MODY, maturity-onset diabetes of the young; N/A, not available; T1DM, type 1 diabetes mellitus.

## DISCUSSION

We reported herein the cases of three patients with *HNF1A, HNF1B*, and *KCNJ11* MODY. The characteristics of these cases are summarized in Table 4. All three patients presented during adolescence with classic features of diabetic ketoacidosis that were initially mistaken for T1DM. Although the cases had atypical presentations, a detailed family history of all three patients was helpful in indicating the possibility of MODY, which was confirmed by molecular testing. The possibility of properly establishing the diagnosis of MODY had a substantial impact on the patients’ treatment and follow-up.

**Table 4:** Clinical characteristics of the patients in all three cases at the time of diagnosis of hyperglycemia

Characteristics	Case 1	Case 2	Case 3
Age at onset	10 years, 9 months	13 years, 3 months	14 years, 6 months
Sex	Female	Female	Male
Parents’ consanguinity	Nonconsanguineous	Nonconsanguineous	Nonconsanguineous
Family history	Diabetes in mother and maternal grandmother	Diabetes in both parents and maternal grandfather	Diabetes in both parents and paternal grandmother
Height	148 cm (z-score 0.69)	119 cm (z-score -5.0)	159 cm (z-score -0.42)
Weight	37.5 kg (z-score 0.26)	19 kg (z-score -4.4)	49 kg (z-score -0.14)
BMI	17.12 kg/m^2^ (z-score 0.04)	13.19 kg/m^2^ (z-score -2.2)	19 kg/m^2^ (z-score 0.10)
C-peptide	0.68 ng/mL (concomitant PG level 210 mg/dL and normal renal function), compatible with maintained pancreatic insulin secretion	0.3 ng/mL (concomitant PG 196 mg/dL and normal renal function)	1.2 ng/ml (concomitant PG level 210 mg/dL and normal renal function), compatible with maintained pancreatic insulin secretion
GAD antibody	Negative	Negative	Negative
HbA1c	13.2%	15.6%	13.5%
Initial diagnosis	T1DM with DKA	T1DM with DKA	T1DM with DKA
Initial treatment	Insulin 0.8 U/kg/day	Insulin 1 U/kg/day	Insulin 0.8 U/kg/day
Other associated findings	N/A	Short stature, abnormal renal function; on abdominal ultrasound, contracted kidneys bilaterally with a parenchymal cyst in the right kidney and unicornuate uterus	Weight gain, acanthosis nigricans
Clues towards MODY	Age at onset, family history, negative GAD antibodies, normal C-peptide levels, poor glycemic control with insulin	Age at onset, family history, short stature, renal dysgenesis, uterine malformation, negative GAD antibodies	Age at onset, family history, very low dose insulin requirement, negative GAD antibodies, normal C-peptide levels
Genetics	Novel heterozygous duplication variation *HNF1A*(NM_000545.8):c.1733dupT (p.Gln579AlafsTer70) in the *HNF1A* gene in the proband and her mother but not in the sibling or father	Novel heterozygous variation *HNF1B*(NM_000458.4):c.458A>G (p.His153Arg) in the *HNF1B* gene in the proband and her mother	Heterozygous variation *KCNJ11*(NM_000525.4):c.853G>A (p.Val285Ile) in the *KCNJ11* gene the proband and his father
Final diagnosis	*HNF1A* MODY	*HNF1B* MODY	*KCNJ11* MODY
Change in treatment	Sulfonylureas	No change	Sulfonylureas
Response to treatment	Achieved good glycemic control (HbA1c 7.2%)	Achieved good glycemic control (HbA1c 6.8%) but progressed to CKD	Achieved good glycemic control (HbA1c 6.9%)

Abbreviations: BMI, body mass index; CKD, chronic kidney disease; DKA, diabetic ketoacidosis; GAD, glutamic acid decarboxylase; HbA1c, glycated hemoglobin; MODY, maturity-onset diabetes of the young; N/A, not available; PG, plasma glucose; T1DM, type 1 diabetes mellitus.

Characterized by an autosomal dominant mode of inheritance, MODY represents a heterogeneous group of monogenic diabetes. Patients with MODY are frequently misdiagnosed with either T1DM or T2DM. Indeed, it is estimated that around 80% of patients with MODY are misdiagnosed with T1DM or T2DM, and a delay of up to 10 years from diabetes presentation to confirmation by molecular diagnosis has been reported (10,11). A diagnosis of MODY should be considered in persons with diabetes without characteristics of T1DM and T2DM who have a family history of diabetes in one parent and/or a first-degree relative of that affected parent. In patients presenting T1DM-like manifestations, the (A) absence of islet autoantibodies (especially, if checked at diagnosis) and (B) preserved beta-cell function (with low insulin requirements and detectable C-peptide in blood or urine) over an extended partial remission phase (at least 5 years after diagnosis) should prompt a genetic testing to rule out MODY. In patients presenting with T2DM-like manifestations, the (A) lack of consistent severe obesity among affected family members and (B) absence of consistent acanthosis nigricans and/or other markers of metabolic syndrome (*e.g.*, hypertension, low HDL-cholesterol, etc.) among affected family members in the presence of a family history of diabetes in two consecutive generations should prompt genetic testing for MODY.

All three cases described herein had atypical presentations, with classic features of diabetic ketoacidosis. Cases of MODY presenting with ketosis are very rare but have been reported. Pruhova and cols. (12) have reported two cases of diabetic ketoacidosis in patients with MODY and *HNF1A* mutations linked to severe dehydration. Similarly, Mcquade and cols. (13) reported a case of diabetic ketoacidosis in a patient with genetically confirmed *HNF1A* MODY. Notably, GAD antibodies – the most frequently occurring pancreatic beta-cell autoantibody found in T1DM – were negative in all three cases, and levels of C-peptide – a marker of endogenous insulin production cosecreted with insulin – were compatible with persistent pancreatic insulin secretion, which is unlikely in cases of T1DM. On the other hand, large cohorts in which MODY has been described with very few occurrences of ketoacidosis or absence of this complication have been documented and referenced (14-16). Of note, diabetic ketoacidosis was considered an exclusion criterion for the diagnosis of MODY in the 2009 ISPAD guideline; this was modified in the 2014 ISPAD updated guideline, as more case reports have highlighted the occurrence of diabetic ketoacidosis in cases of MODY (17,18).

The proband in Case 1 presented to us during adolescence with classic features of diabetic ketoacidosis, favoring a diagnosis of T1DM, and was started on insulin therapy. In view of great variability in plasma glucose levels despite adherence to treatment, C-peptide levels showing insulin secretion (which is not compatible with T1DM), absence of GAD antibodies, and, most importantly, a strong family history of diabetes in the mother and maternal grandmother, the diagnosis of MODY was suspected and confirmed as *HNF1A* MODY by molecular testing. The proband in Case 2 also presented to us during adolescence with ketoacidosis, short stature, abnormal renal function, bilateral contracted kidneys, a simple renal cyst in the right kidney, unicornuate uterus, and a strong family history of diabetes in the mother and grandfather in the third decade of life. A diagnosis of *HNF1B* MODY was suspected and confirmed by molecular testing. The proband in Case 3 was also initially diagnosed with T1DM and was started on insulin therapy, as he presented with classic features of diabetic ketoacidosis. However, in view of multiple episodes of hypoglycemia, even with very low doses of insulin, weight gain, and the appearance of acanthosis nigricans during follow-up, the possibility of T2DM or MODY was considered. On further evaluation, the absence of GAD antibodies, C-peptide levels showing insulin secretion (which is not compatible with T1DM), and, most importantly, a strong family history of diabetes in the father and paternal grandmother pointed toward the diagnosis of MODY, and *KCNJ11* MODY was confirmed by molecular testing. The probands in Cases 1 and 2 had normal BMI. However, the proband in Case 3 presented during follow-up with progressive weight gain, increasing BMI (from 19 kg/m^2^ [25^th^-50th percentile] to 21.6 kg/m^2^[adult equivalent BMI greater than 23 kg/m^2^, *i.e.*, overweight]), and development of acanthosis nigricans. Notably, T2DM often presents around puberty, and the majority of the affected patients are obese. Although obesity is not a feature of MODY, obesity has currently become so common in childhood that children and adolescents with MODY may also develop obesity, with the distinction between T2DM and MODY in these cases becoming challenging (1). Therefore, obesity alone should not preclude genetic testing, especially if the patient’s family history strongly suggests autosomal dominant inheritance of diabetes, or if some affected family members are not obese and do not have other features of metabolic syndrome.

In conclusion, patients with MODY can be easily misdiagnosed as having T1DM or T2DM. Accurately diagnosing MODY through molecular testing will help implement the appropriate treatment plan. This also helps in predicting the risk of complications, which is of fundamental clinical importance for the patients and their families. Hence, in the presence of atypical characteristics in patients with T1DM or T2DM, a careful family history is critical. Obesity and diabetic ketoacidosis have also been reported among MODY subtypes and impose further challenge since they are not part of the original description of MODY by Tattersall & Fajans (19). It is always prudent to maintain a heightened awareness of the possibility of MODY when encountering a patient with a family history of diabetes spanning two consecutive generations. In these cases, molecular testing is advisable to confirm or rule out this diagnosis.
